# Latent variables may be useful in pain’s assessment

**DOI:** 10.1186/1477-7525-12-13

**Published:** 2014-01-30

**Authors:** Donald R Royall, Ricardo Salazar, Raymond F Palmer

**Affiliations:** 1 Department of Psychiatry, The University of Texas Health Science Center At San Antonio, 7703 Floyd Curl Drive MC 7792, San Antonio, TX 78229, USA; 2 Department of Medicine, The University of Texas Health Science Center At San Antonio, 7703 Floyd Curl Drive MC 7792, San Antonio, TX 78229, USA; 3 Departments of Family & Community Medicine, The University of Texas Health Science Center At San Antonio, 7703 Floyd Curl Drive MC 7792, San Antonio, TX 78229, USA; 4 South Texas Veterans Health Administration Geriatric Research Education and Clinical Care (GRECC), The University of Texas Health Science Center At San Antonio, 7703 Floyd Curl Drive MC 7792, San Antonio, TX 78229, USA

**Keywords:** Aging, Assessment, Depression, Pain

## Abstract

**Background:**

Unobserved “latent” variables have the potential to minimize “measurement error” inherent to any single clinical assessment or categorical diagnosis.

**Objectives:**

To demonstrate the potential utility of latent variable constructs in pain’s assessment.

**Design:**

We created two latent variables representing depressive symptom-related pain (Pd) and its residual, “somatic” pain (Ps), from survey questions.

**Setting:**

The Hispanic Established Population for Epidemiological Studies in the Elderly (H-EPESE) project, a longitudinal population-based cohort study.

**Participants:**

Community dwelling elderly Mexican-Americans in five Southwestern U.S. states. The data were collected in the 7th HEPESE wave in 2010 (N = 1,078).

**Measurements:**

Self-reported pain, Center for Epidemiological Studies Depression Scale (CES-D) scores, bedside cognitive performance measures, and informant-rated measures of basic and instrumental Activities of Daily Living.

**Results:**

The model showed excellent fit [χ^2^ = 20.37, DF = 12; p = 0.06; Comparative fit index (CFI) = 0.998; Root mean statistical error assessment (RMSEA) = 0.025]. Ps was most strongly indicated by self-reported pain-related physician visits (r = 0.48, p ≤0.001). Pd was most strongly indicated by self-reported pain-related sleep disturbances (r = 0.65, p <0.001). Both Pd and Ps were significantly independently associated with chronic pain (> one month), regional pain and pain summed across selected regions. Pd alone was significantly independently associated with self-rated health, life satisfaction, self-reported falls, Life-space, nursing home placement, the use of opiates, and a variety of sleep related disturbances. Ps was associated with the use of NSAIDS. Neither construct was associated with declaration of a resuscitation preference, mode of resuscitation preference declaration, or with opting for a “Do Not Resuscitate” (DNR) order.

**Conclusion:**

This analysis illustrates the potential of latent variables to parse observed data into “unbiased” constructs with unique predictive profiles. The latent constructs, by definition, are devoid of measurement error that affects any subset of their indicators. Future studies could use such phenotypes as outcome measures in clinical pain management trials or associate them with potential biomarkers using powerful parametric statistical methods.

## Introduction

The experience of pain is a complex mental phenomenon only partly explained by physical injury or dysfunction. Its assessment can be challenging to clinicians, especially in the elderly, cognitively impaired persons, and minority populations. Cultural bias, emotional state, including anxiety and/or depression, and cognitive resources all contribute to pain’s perception and behavioral manifestations.

Dementia and depression are likely to influence pain’s report in multiple ways. Both conditions have been recently associated with functional central nervous system (CNS) connectivity, especially in the Default Mode Network (DMN) [1]. The DMN is involved in a variety of self-reported tasks and may mediate self-awareness [2]. DMN connectivity is diminished in Alzheimer’s disease (AD), even at pre-clinical stages [3], and abnormally increased in major depression [4,5]. This may explain the poor correlations between cognitive performance and pain reports in dementia, and increased pain complaints in depressive states [6].

We recently developed a novel latent variable approach to address the similar challenges to cognition’s assessment. Using a structural equation modeling (SEM) approach, we explicitly distinguish “dementia-relevant” variance in observed clinical and physiological measures from variance in the observed data that is unrelated to dementia [7]. Latent variable assessment offers many potential advantages over traditional diagnostic methods [8]. First, composite factor scores extracted from the latent variable’s factor loadings can be used as continuously distributed phenotypes. This allows us effectively to rank order individual cases along a syndrome’s continuum and to use powerful parametric statistical methods to find the constructs’ biomarkers. Second, the resulting clinical phenotypes are arguably free of cultural, linguistic or educational bias.

A further advantage of our approach is that the latent variables are modular, and easily adapted to other clinical problems. In this analysis, we use SEM to parse the observed variance in self-reported pain complaints into two compartments: Depressive symptom-related pain (Pd) and “Somatic” pain (Ps). Future studies may be able to test the biomarkers of these constructs and/or use them as outcomes in pharmacological studies of pain’s management.

## Methods

The data were collected in Wave 7 of the Hispanic Established Population for the Epidemiological Study of the Elderly (H-EPESE) study. The institutional review board of the University of Texas Medical Branch at Galveston, Texas approved the H-EPESE project. All individuals discussed the study with trained research staff and provided written informed consent.

### Participants

The H-EPESE cohort was originally established ca 1993–94 with a representative sample of 3,050 Mexican Americans aged 65 and over and residing in five Southwestern states (i.e., Texas, New Mexico, Colorado, Arizona, and California). These subjects were re-examined in 1995–96 (N = 2438), 1998–99 (N = 1980), 2000–01 (N = 1683), and 2004–05 (N = 1167). An additional 902 Mexican Americans aged 75 and over were added in 2004–05. The combined sample of 2067 was followed up approximately 2 ½ years later during 2007 (N = 1,542 subjects then aged 78 and over). Data was collected on the 7th wave in 2010 (N = 1,078). An 8th wave of follow-up is currently in progress. In recent waves, investigators have contacted and interviewed in-person a close family member, usually an adult child, to detail participants’ current health care needs and family and financial situations.

#### Clinical variables

The H-EPESE Manual of Procedures is updated at each Wave. All procedures are available in Spanish translation.

A. Pain: Pain was assessed by subject self-report. Table 1 lists the specific pain assessments employed, including: Has pain restricted your daily activities in last 12 months? (PADL); Have you seen a doctor about pain? (PMD); Are you taking medication for pain? (PMED); and How much has pain interfered with your sleep in last 12 months? (PSLP). PADL and PSLP were assessed on a three point Likert Scale ranging from “A lot” to “Not at all”. PMD and PMED were assessed dichotomously as “Yes” or “No”.

**Table 1:** **Variables used for SEM model (n** = **1079, missing n** = **69)**

**Indicators**	
CES-D (Mean/SD)	10.7 (9.2)
Pain restricted daily	
activities in last 12	
months? (PADL)	
% “A lot”	17.6%
% “Some”	23.7%
% “Not at all”	58.6%
Seen Dr. about	
Pain? (PMD)	
% “Yes”	42.3%
Taking Medication for Pain? (PMED)	
% “Yes”	37.6%
Pain restricted sleep in last 12 months (PSLP)	
% “A lot”	7.5%
% “Some”	17.5
% “Not at all”	75%
**Covariates**	
Number of pain medications (NpainRx)	
Live alone	See Table 2
Age	
Gender	
BMI	
Income	
MMSE	

B. Physical Function: Functional status in the H-EPESE is assessed using measures of Basic Activities of Daily Living (BADL) [9] and Instrumental Activities of Daily Living (IADL) [10].

C. Cognition and Mood: The main assessment of cognitive status employed in the H-EPESE has been the Mini-Mental State Exam (MMSE) [11]. In Wave 3, an executive measure was added (i.e., CLOX: An Executive Clock-Drawing Task) [12]. In wave 7 the Neuropsychiatric Inventory (NPI) [13] was added. The Center for Epidemiologic Studies Depression scale (CES-D) [14] is available at all Waves.

D. Living Arrangements: At each wave H-EPESE obtains a complete census of all individuals living in the household including their relationship to the subject and all subjects are traced to nursing homes or assisted living sites using an abbreviated questionnaire.

E. Health Care Service Utilization: H-EPESE collects information on health care service utilization [15]. Respondents who reported having been diagnosed with cardiovascular problems, stroke, and cancer are also asked about hospitalizations related specifically to those problems.

F. Medical Conditions: H-EPESE includes self-reported/informant rated measures of chronic medical conditions, including hypertension, diabetes, cancer, heart disease, stroke, hip fracture, arthritis, urinary and bowel incontinence, and other problems.

G. Anthropometric measures: Obesity is measured by body mass index (BMI), and waist circumference.

#### Specific clinical assessments

CLOX: An Executive Clock-Drawing Task) [12]: CLOX is a two-part clock-drawing task divided into a non-prompted “executive” version (CLOX1) and a prompted “constructional” version (CLOX2). Both tasks are scored on the same 15 point metric. Thresholds for “impairment” are set to young adult norms at CLOX1 = 10/15 and CLOX2 = 12/15. Using these thresholds, a CLOX rated “dementia-type” can be estimated [16].

The Center for Epidemiologic Studies Depression scale (CES-D) [14]: The CES-D has been used extensively with older populations [17], including Mexican Americans [18]. It is typically used as a continuous variable measuring depressive symptomatology or psychological distress or as a dichotomous variable with a score of 16+ indicating high levels of depressive symptomatology and suggesting possible clinical depression.

The Mini-Mental Status Exam (MMSE) [11]: The MMSE is a well known and widely used test for screening cognitive impairment [19]. Scores range from 0 to 30. Scores less than 24 reflect cognitive impairment.

The Neuropsychiatric Inventory (NPI) [13]: The NPI assesses ten behavioral disturbances occurring in dementia patients: delusions, hallucinations, dysphoria, anxiety, agitation/aggression, euphoria, disinhibition, irritability/lability, apathy, and aberrant motor activity. The NPI uses a screening strategy to minimize administration time, examining and scoring only those behavioral domains with positive responses to screening questions. Both the frequency and the severity of each behavior are determined. Information for the NPI is obtained from an informant who is familiar with the subject’s behavior.

Descriptive statistics are presented in Table 2. The Wave 7H-EPESE sample is elderly (mean age 85.7 years) and poorly educated (mean 5.0 years of formal education). There is a slight female majority. 54.3% were U.S. born. 81.8% were interviewed in Spanish. 35.1% of respondents reported an annual income < $10,000.

**Table 2:** **Characteristics of the wave 7 HEPESE sample (N** = **1,078)**

**Age (mean/SD)**	**85.6 (4.01)**
Female (%)	65
US Born (%)	54.3
Interview in Spanish (%)	81.8
Married (%)	31.4
Live Alone (%)	30.3
Annual Income < $10,000 (%)	35.1
Medicare insured (%)	96.7
Body Mass Index (mean/SD)	27.5 (5.6)
MMSE Score (mean)	21.2 (7.5)
Any BADL Impairment (%)	49.5
Any IADL Impairment (%)	85.7
**Health Conditions**	
Heart attack (ever;%)	10.0
Stroke (%)	10.6
Cancer (ever;%)	10.6
Diabetes (ever;%)	36.5
Hip fracture (%)	7.1
Arthritis (ever;%)	65.9
Pain or discomfort (In the past month;%)	84.2
Hypertension (Self-report & measured;%)	74.1
Number of Pain Medications (Mean/SD)	0.37 (0.56)
**Interview Type**	83.1
In Person Interview (%)	
Assisted Proxy (%)	7.7
Proxy (%)	9.2

BADL = Basic Activities of Daily Living; CES-D = Center for Epidemiological Studies-Depression scale; H-EPESE = Hispanic Established Population for Epidemiological Studies in the Elderly; IADL = Instrumental Activities of Daily Living; MMSE = Mini-Mental State Exam; US = United States.

On average, the H-EPESE sample is characterized by low normal cognitive performance, given their advanced age, and low educational attainment. Of the 1078 respondents, 224 (20.8%) scored < 18/30 on the MMSE, i.e., below its recommended threshold for cognitive impairment in this demographic [20]. The cohort exhibited a subsyndromal depressive symptom burden on the CES-D. 146 respondents (13.5%) scored above a conservative “clinically significant” threshold of 20/60. A high fraction of subjects report potentially significant medical problems, including heart attack (10%) stroke (10.6%), cancer (10.6%), diabetes (36.5%), hip fractures (7.1%) arthritis (65.9%) or hypertension (74.1%).

### Statistical analysis

**Figure 1:**
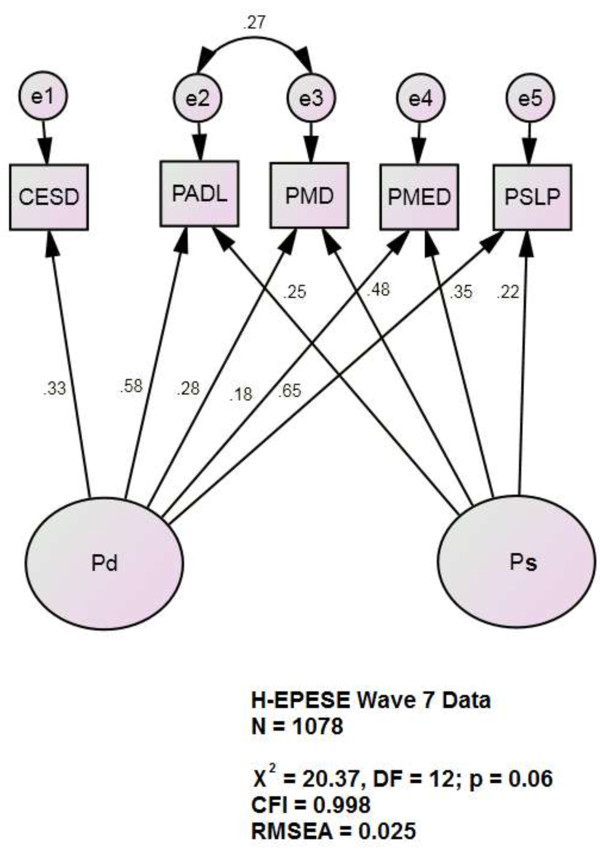
Latent variables Pd and Ps*. CES-D = Centers for Epidemiological Studies-Depression scale; CFI = Comparative Fit Index; DF = degrees of freedom; H-EPESE = Hispanic Established Population for Epidemiological Studies in the Elderly; PADL = self reported pain-related functional limitations; Pd = depression-related pain report; PMD = self reported pain-related doctor visits; PMED = self reported pain-related medication use; Ps = somatic-related pain report; RMSEA = Root Mean Statistical Error Assessment; PSLP = self-reported pain-related sleep disturbance. *All observed variables are adjusted for age, gender, the number of prescribed pain medications, Mini-mental State Exam scores, body mass index, living alone and adequacy of monthly financial support (covariates not shown for clarity).

The descriptive statistical analysis was performed in STATISTICA, version 10 for Windows (Statsoft, Inc., Tulsa, OK, U.S— http://www.statsoft.com). SEM analysis was performed using Analysis of Moment Structures (AMOS) [21]. Data were fit to a two factor latent variable model (see Figure 1) and compared to a single factor model with four pain items and one depression item as indicators of the latent variable. The bi-factor model uses CES-D scores as the “target” indicator of a second factor. This effectively parses the variance shared across the pain indicators into to factors. The first represents the variance shared with depressive symptoms (i.e., “Pd”). The second represents the shared variance that is explicitly not shared with depressive symptoms. We interpreted this as “somatic” pain (i.e., “Ps). This approach is modular. Any alternative target indicator can be substituted for CES-D scores in Figure 1 and the variance shared across the pain indicators would be parsed again.

The latent variables were scaled by fixing the means to 0 and the variances at 1, with all loadings freely estimated. Residual variances were uncorrelated with one exception. The residual covariance between *pain restricting daily activities* (PADL) and *taking medication related to pain* (PMD) was estimated based on modification indices in AMOS. This significantly improved model fit and is theoretically reasonable. Parameter estimation was obtained by maximum likelihood. The two latent variables (Pd and Ps) were extracted. Seven covariates: age, gender, income, number of pain medications, living alone status, Body Mass Index (BMI) and MMSE scores, were used to adjust the latent variables’ indicators and hence the latent constructs themselves (see Tables 1 and 2 for description of items and covariates). Ps and Pd were validated by simultaneously regressing them onto 36 clinical outcomes in separate multivariate regression models. Both the latent variables’ indicators and the dependent outcome variable of interest were adjusted for all seven covariates. Bonferonni correction was used to adjust for multiple comparisons with a p value of 0.002 considered statistically significant. Mutlivariate normality was assessed using Mardia’s coefficient [22].

*Missing data:* AMOS uses Full Information Maximum Likelihood (FIML) methods to address missing data. FIML uses the entire observed data matrix to estimate parameters with missing data. In contrast to list-wise or pair-wise deletion, FIML yields unbiased parameter estimates and preserves the overall power of the analysis. Along with multiple imputation approaches, FIML is recommended as one of the best approaches to handling missing data [23,24].

*Fit Indices:* The validity of structural models was assessed using common fit indices. A non-significant chi-square signifies that the data are consistent with the model [25]. The comparative fit index (CFI), with values ranging between 0 and 1, compares the specified model with a model of no change [26]. CFI values below 0.95 suggest model misspecification. Values of 0.95 or greater indicate adequate to excellent fit. A root mean square error of approximation (RMSEA) of 0.05 or less indicates a close fit to the data, with models below 0.05 considered “good” fit.” [27]. All three fit statistics should be simultaneously considered to assess the adequacy of the models to the data.

## Results

Table 1 presents the data used to construct the latent variables. 527 respondents (48.9%) endorsed pain in the last month. 256 (23.8%) reported “some” and 190 (17.6%) reported “a lot” of pain-related functional limitations. 456 (42.3%) reported seeking medical treatment for pain. 402 (37.3%) reporting taking one or more prescribed pain medications (mean 0.37 ± 0.56). 188 (17.4%) reported “some” pain-related sleep disturbances. 81 (7.5%) reported “a lot”.

Table 3 presents the base SEM model’s regression coefficients. Also shown are the coefficients of the association between the model indicators and covariates. The assumption of multivaraite normality was not met in this data sample, we therefore utilized a bootstrap method to obtain unbiased estimates and standard errors. All indicators loaded significantly on Pd and Ps, independently of the covariates. Only Pd was allowed to be indicated by CES-D scores (loading = 0.33, p ≤0.001). Ps was most strongly loaded by self-reported pain-related physician visits (PMD) (r = 0.48, p ≤0.001). Pd was most strongly loaded by self-reported pain-related sleep disturbances (PSLP) (r = 0.65, p <0.001). Ps was least strongly loaded by PSLP (r = 0.23, p = 0.006).

**Table 3:** Base SEM model parameters with covariates

			**Estimate**	**S.E.**	**p**	**Standardized estimate**
			**Pd Loadings**			
CES-D	<−−−	Pd	2.999	0.339	<0.001	0.327
PADL	<−−−	Pd	0.447	0.038	<0.001	0.580
PMD	<−−−	Pd	0.137	0.033	<0.001	0.277
PMED	<−−−	Pd	0.087	0.024	<0.001	0.180
PSLP	<−−−	Pd	0.396	0.031	<0.001	0.650
			**Ps Loadings**			
PADL	<−−−	Ps	0.191	0.060	0.001	0.248
PMD	<−−−	Ps	0.239	0.048	<0.001	0.483
PMED	<−−−	Ps	0.171	0.035	<0.001	0.354
PSLP	<−−−	Ps	0.137	0.050	0.006	0.225
			**Covariate Associations**			
CES-D	<−−−	Age	−0.060	0.071	0.398	−0.026
PADL	<−−−	Age	−0.003	0.005	0.595	−0.014
PMD	<−−−	Age	−0.002	0.003	0.509	−0.016
PMED	<−−−	Age	−0.001	0.002	0.512	−0.012
PSLP	<−−−	Age	−0.008	0.005	0.076	−0.054
CES-D	<−−−	BMI	−0.017	0.054	0.746	−0.011
PADL	<−−−	BMI	0.008	0.004	0.043	0.056
PMD	<−−−	BMI	0.002	0.002	0.395	0.022
PMED	<−−−	BMI	0.002	0.002	0.143	0.028
PSLP	<−−−	BMI	0.008	0.004	0.020	0.076
CES-D	<−−−	Gender	2.811	0.573	<0.001	0.146
PADL	<−−−	Gender	−0.006	0.039	0.888	−0.003
PMD	<−−−	Gender	−0.027	0.024	0.257	−0.026
PMED	<−−−	Gender	0.019	0.017	0.278	0.018
PSLP	<−−−	Gender	0.037	0.037	0.313	0.029
CES-D	<−−−	Income	0.345	0.754	0.647	0.015
PADL	<−−−	Income	0.050	0.051	0.331	0.026
PMD	<−−−	Income	0.012	0.031	0.694	0.010
PMED	<−−−	Income	0.017	0.022	0.441	0.014
PSLP	<−−−	Income	0.119	0.048	0.013	0.078
CES-D	<−−−	Live alone	1.698	0.599	0.005	0.085
PADL	<−−−	Live alone	0.033	0.041	0.426	0.019
PMD	<−−−	Live alone	0.041	0.025	0.098	0.038
PMED	<−−−	Live alone	0.018	0.018	0.321	0.017
PSLP	<−−−	Live alone	0.014	0.038	0.712	0.011
CES-D	<−−−	MMSE	−0.446	0.048	<0.001	−0.290
PADL	<−−−	MMSE	−0.014	0.003	<0.001	−0.110
PMED	<−−−	MMSE	−0.003	0.001	0.042	−0.037
PMD	<−−−	MMSE	−0.001	0.002	0.483	−0.017
PSLP	<−−−	MMSE	−0.002	0.003	0.577	−0.017
CES-D	<−−−	NpainRx	2.539	0.484	<0.001	0.155
PADL	<−−−	NpainRx	0.823	0.033	<0.001	0.599
PMD	<−−−	NpainRx	0.591	0.020	<0.001	0.671
PMED	<−−−	NpainRx	0.715	0.014	<0.001	0.832
PSLP	<−−−	NpainRx	0.372	0.031	<0.001	0.343

Model fit: χ^2^ = 20.4 (df = 12, p = 0.06); CFI = 0.998; RMSEA = 0.025.

Alone = living alone; BMI = calculated body mass index; CES-D = Centers for Epidemiological Studies-Depression scale; Income = informant assessment of monthly income adequacy; MMSE = Mini-Mental State Exam; NpainRx = number of prescribed pain medications; PADL = self-reported pain-related functional limitations; Pd = depression-related pain report; PMD = self-reported pain-related doctor visits; PMED = self-reported pain-related medication use; Ps = somatic-related pain report; PSLP = self-reported pain-related sleep disturbance.

This two factor model demonstrated far better fit [χ^2^ = 20.4 (df = 12, p = 0.06); CFI = 0.998; RMSEA = 0.025] than a single factor model of pain items with depression [χ^2^ = 126.5 (df = 16, p = 0.0001]; CFI = 0.975; RMSEA = 0.08). The two factors are orthogonal by design.

Table 4 presents the results of several multivariate regression models of selected clinical outcomes. Pd alone was significantly independently associated with self-rated health and life satisfaction. Both Pd and Ps were significantly independently associated with chronic pain (> one month), regional pain, and pain summed across selected regions. Both were significantly and independently associated with a history of arthritis, whether reported by the respondent or confirmed by the informant.

**Table 4:** Multivariate regression models of Ps and Pd as predictors of selected clinical outcomes

**Model number**	**Dependent variable**	**Pd standardized regression coefficient**	**p**	**Ps standardized regression coefficient**	**p**	**Total R**^**2**^	**R**^***2 ***^**(covariates only)**
**1**	**Chronic pain**	0.25	≤0.001	0.32	≤0.001	0.30	0.02
**2**	**Pain “all over”**	−0.32	≤0.001	0.11	0.04	0.19	0.08
**3**	**Legs**	−0.35	≤0.001	0.24	≤0.001	0.40	0.22
**4**	**Feet**	−0.25	≤0.001	0.16	0.006	0.26	0.18
**5**	**Knees**	−0.33	≤0.001	0.22	≤0.001	0.38	0.22
**6**	**Hip**	−0.29	≤0.001	0.12	0.02	0.24	0.14
**7**	**Back**	−0.22	≤0.001	−0.20	≤0.001	0.25	0.16
**8**	**Summed pain**	−0.42	≤0.001	0.25	≤0.001	0.58	0.34
**9**	**Alzheimer’s (r)**	−0.07	0.107	−0.16	≤0.001	0.06	0.03
**10**	**Cancer (r)**	0.19	≤0.001	−0.05	0.38	0.16	0.11
**11**	**Arthritis (r)**	−0.16	≤0.001	0.15	0.001	0.14	0.09
**12**	**CA (i)**	0.03	0.42	0.02	0.67	0.01	0.01
**13**	**Arthritis (i)**	−0.10	0.03	0.15	0.002	0.10	0.07
**14**	**Both (i)**	−0.09	0.04	0.12	0.02	0.07	0.05
**15**	**Opiate use**	−0.014	≤0.001	0.06	0.17	0.25	0.23
**16**	**NSAID use**	0.05	0.20	0.09	0.03	0.03	0.02
**17**	**Health**	−0.38	≤0.001	0.02	0.76	0.20	0.05
**18**	**QOL**	−0.47	≤0.001	0.00	0.99	0.25	0.02
**19**	**Life Space**	0.10	0.02	0.04	0.32	0.09	0.07
**20**	**Sleep Disturbances (Total)**	0.55	≤0.001	0.02	0.71	0.33	0.03
**21**	**Early wakening**	−0.36	≤0.001	0.07	0.19	0.18	0.05
**22**	**Frequent wakening**	−0.17	≤0.001	0.11	0.02	0.07	0.03
**23**	**Feeling tired**	−0.44	≤0.001	0.10	0.07	0.27	0.06
**24**	**Agitation (summed)**	−0.19	≤0.001	0.01	0.88	0.05	0.01
**25**	**NPI Nighttime agitation**	−0.17	≤0.001	0.02	0.68	0.04	0.01
**26**	**NPI restless**	−0.05	0.23	0.01	0.88	0.00	0.00
**27**	**NPI impatient**	−0.05	0.19	0.00	0.95	0.02	0.02
**28**	**NPI Δ weight**	−0.08	0.06	0.02	0.62	0.02	0.01
**29**	**NPI resistive**	−0.09	0.03	0.03	0.54	0.04	0.03
**30**	**NPI sad**	−0.18	≤0.001	0.01	0.77	0.06	0.02
**31**	**5 chair stands**	0.03	0.58	0.08	0.14	0.04	0.03
**32**	**Stands safely**	0.19	≤0.001	−0.05	0.38	0.16	0.11
**33**	**Falls (i)**	−0.07	0.11	−0.02	0.71	0.02	0.01
**34**	**Falls (r)**	−0.32	≤0.001	0.00	0.99	0.19	0.09
**35**	**Fear of falling**	−0.29	≤0.001	0.00	0.97	0.16	0.08
**36**	**Nursing Home**	−0.08	0.04	−0.07	0.12	0.02	0.01
**37**	**DNR declaration**	0.02	0.66	0.04	0.43	0.01	0.01
**38**	**DNR mode**	0.00	0.93	0.01	0.83	0.01	0.01
**39**	**DNR**	0.03	0.53	0.05	0.40	0.01	0.00

Pd = Depression related pain independent predictor variable; Ps = Somatic related pain independent predictor variable; Number of pain medications (NPainmeds); Model Covariates: Live alone, age, gender, BMI, Income, MMSE; CA = cancer; DNR = Do Not Resusitate order; (i) = informant rated; NPI = Neuropsychiatric Inventory; NSAID = non-steriodal anti-inflammatory agents; QOL = quality of life; (r) = respondant rated.

Pd was associated with a self-reported h/o cancer, but neither construct was associated with an informant confirmed history of that condition. Similarly, Pd was associated with self-reported falls, and a self-reported fear of falling, but neither construct was associated with informant confirmed falls.

Pd was associated with a variety of sleep-related disturbances, including frequent wakening, early wakening, daytime fatigue, and summed total sleep disturbances. Ps was not associated independently with any sleep disturbance. Neither construct was associated with the ability to successfully complete five chair stands. However, Pd was associated with failure to attempt chair stands.

The use of opiates was uniquely associated with Pd scores, the use of NSAIDS with Ps scores. Pd was associated with Life-space, nursing home placement, and informant ratings of stubbornness and sadness. Ps was not associated with any of these four outcomes. Neither construct was associated with declaration of a resuscitation preference, mode of resuscitation preference declaration, or with opting for a “Do Not Resuscitate” (DNR) order.

Most of the significant effects were modest and added < 20% of additional variance to the model. However, Pd’s associations with self-rated health, sleep disorders, and life satisfaction were exceptionally strong.

## Discussion

This analysis illustrates the potential of latent variables to parse observed data into “unbiased” constructs with unique predictive profiles. The latent constructs, by definition, are unbiased by measurement error that affects any subset of their indicators. Such error is “residual” to the latent variable(s) of interest. However, latent constructs do have quantifiable statistical error parameters, and are not technically “error free”.

Huber et al. [28] have previously attempted to build a comprehensive model of pain processing in an SEM format. Our approach differs subtly from theirs in that it effectively parses the variance into target-related and unrelated fractions. This is a methodological advance. The resulting models have improved fit, and fewer intercorrelated residuals. The resulting latent variables are orthogonal to each other and, like their dementia-related counterparts [7] can be output as continuously varying subjective “pain” phenotypes. Our method could easily be adapted for the extraction of a more comprehensive set of latent constructs had such measures been available. However, we were constrained by H-EPESE’s psychometric battery, which did not include anxiety or other relevant mood states.

Our analysis provides face and discriminant validity for the modeled constructs. Ps was uniquely associated with NSAID use. Pd was uniquely associated with informant-rated dysphoria, self-reported anxiety, and self-reported sleep disturbances that are indicative of depressive illness (e.g., early morning wakening).

Pd’s unique association with a broad range of self-reported sleep disturbances was striking. Insomnia is a risk factor for incident depression [29,30] and both sleep disturbance and depressive symptoms are frequently reported in musculoskeletal pain syndromes [31-33]. This suggests that the report of pain-related sleep disturbances should prompt a careful review of depressive symptoms and may warrant anti-depressant therapy.

Pd is most strongly indicated by PMED, suggesting either that drug seeking may be related to depressive symptoms [34] or that the depressive component of self-reported pain may be iatrogenic. It has been suggested that persons with lower education and minorities, including Hispanics, are less likely to use mental healthcare services, and more likely to seek help for mood and mental disorders in the medical healthcare system [35,36]. On the other hand, Pd was uniquely significantly associated with opiate use. Post-operative depressive symptoms are a stronger predictor of long-term post operative opiate use than is the severity of post-operative pain [37], and a fraction of those who receive opiates might benefit from anti-depressant intervention, in conjunction with, but possibly instead of opiate analgesics [38].

Pain is often cited as a risk factor for suicidal ideation (SI). Unfortunately, SI is not assessed in H-EPESE, but Pd was a unique predictor of self-rated QOL and health status. Self-rated health and QOL are potent predictors of mortality [39,40] as is major depression [41,42], subsyndromal depressive symptoms [43,44] and depressive personality traits [45]. Pd could potentially mediate their associations with mortality in H-EPESE [46].

It has been reported that pain increases the odds of refusing DNR orders [47]. However, we did not confirm this. The failure of either Pd or Ps to predict DNR status suggests that such advance directives may not derive from pain-related considerations in community dwelling Hispanics [48]. Similarly, we did not find strong associations between these constructs and informant-rated agitation, despite an established literature implicating pain as a cause of behavioral agitation, particularly among demented and institutionalized persons [49].

Depressive symptoms, even subsyndromal depressive symptoms, are potent risk factors for future cognitive decline, dementia conversion and institutionalization. Similarly, Pd, but not Ps, was significantly associated with life space, nursing home placement, and informant ratings of resistiveness, an institutionalization risk factor. Since antidepressant treatment might be able to reduce Pd-related variance in self-reported pain, depressive symptoms should be carefully screened in medical and rehabilitation patients who are slated for institutionalization following recovery from painful procedures [50,51].

Our model does not address potential cognitive determinants of pain perception. H-EPESE has a limited cognitive assessment, consisting only of the MMSE and CLOX, an executive clock-drawing task [12]. CLOX scores are not available from wave 7 at the time of this analysis, and we chose instead to treat MMSE scores as a covariate. However, future analyses may be able to construct a latent cognitive variable from these measures (i.e., “Pc”). Then, the unique contributions of Pc, Pd and Ps to these outcomes could be examined in similar multivariate models.

MMSE, CLOX scores and IADL ratings can also be combined into a latent homolog of “d”, i.e., the cognitive correlates of functional status [52]. d scores have been mapped to elements of the DMN, which is dysfunctional in both depression and Alzheimer’s Disease (AD) [1,52]. The significance of this is that the DMN is involved in a variety of “self-referential” tasks [2] and deactivated by painful visceral stimuli [53]. DMN dysfunction may account for the notable “anosagnosia” seen in AD cases [54]. Anosagnosia refers to diminished insight, and is possibly relevant to a broad range of self-reported mental states, including both depression and pain [55]. Thus, both Ps and Pd may be vulnerable to assessment bias in the setting of DMN-related disorders. This may explain the often noted discrepancy between the pain self-reports of demented persons and their comportment. The DMN is hyperactive in depression [4] and may lead to overestimates of painful stimuli. SEM models offer the potential to adjust pain reports for inter-individual variability in d and by extension, DMN structure.

We were limited in this analysis to categorical and often dichotomously rated pain indicators. Future models could be built from more normally distributed pain ratings, ideally from validated pain assessment scales. The resulting latent variables would be more normally distributed, which would then allow us to associate them with potential biomarkers, using powerful parametric statistical methods. The necessary observed variables could be obtained by clinicians in the field, or over the phone. Pd and Ps scores could then be generated in the field by web or phone-based applications running multivariate regression classification models. Future studies might then use these phenotypes as outcome measures in clinical pain management trials. If we could demonstrate specific treatment effects of analgesic, antidepressant or cognitive enhancing therapies on Ps, Pd (and potentially Pc) respectively, then treatment decisions could be individualized on the basis of an individual’s Ps, Pd and Pc scores.

In summary, a latent variable approach to pain assessment offers an alternative to demonstrably biased self-reported pain assessment. The latent variables Pd and Ps can be constructed in many existing datasets, from ad hoc combinations of pain and depressive symptom ratings. This can allow high quality pain assessments to be made retrospectively in existing datasets or prospectively in minority or rural populations, far from tertiary care centers. Furthermore, Pd and Ps scores represent continuously varying pain phenotypes that can be associated with potential bio-markers, or used as outcomes measures in clinical trials. This may help individualize pain management regimens, especially in minority, rural or cognitively disadvantaged patients.

## Competing interests

Dr. Royall holds a copyright on CLOX. Drs. Royall and Palmer have filed a provisional patent pertaining to the use of latent variables in clinical case-finding. Dr. Salazar declares no conflict of interests.

## Authors’ contributions

DRR: Conception and design; analysis, and interpretation of data; drafting of the manuscript and writing the paper. RS: Drafting and preparation of the manuscript, interpretation of data. RFP: Analysis, and interpretation of data; statistical expertise; supervision. All authors read and approved the final manuscript.
